# Developing a Weight Management and Metabolic Health Program to support patient-centred, effective, and efficient treatment for veterans with overweight or obesity: protocol for a quality improvement programme

**DOI:** 10.1017/S1463423625100650

**Published:** 2026-01-12

**Authors:** Devvrat Malhotra, James Henderson, Cassie D. Turner, Robert W. O’Rourke, Keith B. McAuley, Michele Heisler, Mira Otto, Chelsea Thomason, Jessica L. O’Neill, Katherine Freeman, Marissa W. Dunham, Emily P. Bartley, Andrea Hess, Valerie Kruse, Kathryn Ruttan, Ashlie L. Haeussler, Gabriel Solomon, Christopher Grondin, Paul S. Kim, Richard J. Schildhouse, Adam Tremblay, Dina H. Griauzde

**Affiliations:** 1 VA Ann Arbor Healthcare System, Ann Arbor, MI, USA; 2 Department of Internal Medicine, University of Michigan Medical School, Ann Arbor, MI, USA; 3 University of Michigan Institute for Healthcare Policy and Innovation, Ann Arbor, MI, USA; 4 Department of Learning Health Sciences, University of Michigan Medical School, Ann Arbor, MI, USA; 5 Department of Surgery, University of Michigan, Ann Arbor, MI, USA

**Keywords:** Anti-obesity medications, obesity, veterans, Veterans Health Administration, weight management

## Abstract

**Background:**

Veterans Affairs Medical Centers offer multiple weight-loss treatments, including a comprehensive lifestyle intervention program (i.e., MOVE!), anti-obesity medications (AOMs) and bariatric surgery. Yet, most eligible veterans do not receive these treatments.

**Aim:**

To describe the design, rationale, and planned evaluation of a comprehensive Weight Management and Metabolic Health program (WMMHP), consisting of (1) weight-focused visits with physicians or pharmacists trained in obesity medicine; (2) patient-centered use of available weight-loss treatments; and (3) coordinated, team-based care.

**Methods:**

This is a quality improvement program implemented within the VA Ann Arbor Healthcare System. WMMHP eligibility criteria include body mass index (BMI) ≥ 30 kg/m^2^ or BMI ≥ 27 kg/m^2^ and ≥ 1 weight-related condition and participation in the MOVE! program. We plan to conduct an 18-month retrospective program evaluation using a propensity-matched cohort analysis to estimate the added benefit of WMMHP vs. MOVE! alone. The primary outcome will be mean change in weight at 18 months after baseline. Secondary outcomes will include mean weight loss at 6, 12, and 24 months, percentage of patients achieving thresholds of ≥ 5%, ≥ 10%, and ≥ 15% weight loss, initial prescriptions for and refilled prescriptions as a measure of adherence to AOMs, and referrals to, engagement with, and completion of bariatric surgery. We will also examine between-group differences in health system resource utilization.

**Discussion:**

The WMMHP is an innovative approach to improving treatment and outcomes for veterans with overweight and obesity. If effective, its components may inform obesity care delivery in VA and non-VA settings.

## Background

An estimated 41% of veterans who receive care within the veterans Health Administration (VHA) have obesity (Breland *et al*., 2017). Since 2001, the VHA National Center for Health Promotion and Disease Prevention has prioritized treatment of overweight and obesity through the development, implementation, and dissemination of the MOVE! Weight Management Program (referred to hereafter as MOVE!) (U. D. of V. A. Administration Veterans Health, n.d.). MOVE! is a comprehensive lifestyle intervention that aims to support dietary, physical activity, and behavioural modifications. Additionally, veterans with overweight or obesity may be eligible for treatment with anti-obesity medications (AOMs) and/or bariatric surgery. To date, the full range of weight management treatments (WMTs) are underused within VHA. Specifically, among eligible veterans, less than 10% participate in MOVE!, less than 2% used AOMs between 2008 and 2017, and less than 0.5% undergo bariatric surgery (Semla *et al*., 2017; Hung *et al*., 2024; Gunnar, 2017; Robinson *et al*., 2022; Maciejewski *et al*., 2018; ‘Geographic Variation in Obesity, Behavioral Treatment, and Bariatric Surgery for Veterans – Maciejewski – 2019 – Obesity – Wiley Online Library,’ n.d.). Similarly low rates of WMT utilization occur in non-VA setting (Henderson *et al*., 2024; Campos *et al*., 2020; Alva *et al*., 2022; Montero et al., 2023). These low rates may be due, in part, to primary care clinicians’ limited training in obesity medicine and brief clinic visits with multiple competing priorities (Oshman *et al*., 2023; Kaplan *et al*., 2018; Petrin *et al*., 2017; Mastrocola *et al*., 2020).

In 2016, with the goal of improving treatment and outcomes for veterans with obesity, VHA convened a multidisciplinary group of clinical and research experts in a State-of-the-Art (SOTA) Conference for Weight Management (Masheb *et al*., 2017). Conference attendees recognized that VHA, the largest healthcare system in the United States, is uniquely positioned to improve individual and population health through delivery of coordinated, effective, and patient-centred obesity treatment. To realize this potential, attendees called for the development of system-level strategies to increase preference-sensitive use of all WMTs (Semla *et al*., 2017) and population-based approaches to track patients over time and optimize achievement of clinically relevant weight loss (e.g., ≥5% weight loss) (Raffa *et al*., 2017).

Unfortunately, after nearly a decade, little remains known about how to fully translate SOTA priorities into practice, though the rapidly changing landscape of obesity medicine underscores the critical need for effective strategies. Specifically, the recent availability of new, highly effective incretin mimetics for weight management (i.e., semaglutide, tirzepatide) has accelerated AOM prescribing practices (DeSilver, 2024). Within VHA, AOM prescribing increased by 177% from 2022 to 2023; this increase is largely attributable to prescribing of semaglutide for weight management (Internal VA communication). In May 2024, VA acknowledged this increase as a promising advance in weight management care delivery for veterans but also recognized four potential areas of resource scarcity that could impede use of AOMs among an estimated ∼3 million treatment-eligible veterans (Internal VA communication). These include (1) variable access to incretin mimetics for weight management due to drug supply chain issues, (2) limited access to clinicians to prescribe and support use of AOMs, (3) limited access to MOVE! comprehensive lifestyle intervention, and (4) budgetary constraints (Internal VA communication).

Novel strategies are now urgently needed to address areas of resource scarcity and deliver coordinated, effective, and patient-centred obesity treatment to potentially millions of veterans who may benefit from weight loss. To address this need, in January 2022, we developed and launched a comprehensive Weight Management and Metabolic Health Program (WMMHP) to optimize treatment for veterans with overweight and obesity. WMMHP draws on our team’s prior success in improving obesity care delivery in a non-VA setting (Griauzde et al. 2022; 2024) and consists of three key components: (1) weight-focused visits with physicians or clinical pharmacy practitioners (CPPs) trained in obesity medicine; (2) patient-cented use of AOMs and/or referral to bariatric surgery in conjunction with lifestyle change; and (3) coordinated, team-based care. The aim of this paper is to describe the design, rationale, and planned evaluation of WMMHP. If WMMHP is found to be effective, its components may inform the delivery of high-quality obesity treatment within VA and non-VA settings nationwide.

## Methods

### Study design

This is a clinical quality improvement programme. In this retrospective matched cohort study, we will evaluate key measures of programme implementation (e.g., referrals to WMMHP, veterans’ uptake and engagement once referred) and clinical effectiveness (e.g., weight change). The primary clinical outcome is weight change at 18 months; we will use a matched cohort to evaluate the individual-level weight loss effectiveness of WMMHP versus MOVE! alone. Data will be collected from VA electronic health records (EHR) and databases. The evaluation period for this study is from January 1, 2022, to December 31, 2024. This programme evaluation was approved by the VA Ann Arbor Healthcare System’s (VAAAHS) Quality Management Committee.

### Study setting

The United States is divided into 18 regional systems of VA care, referred to as veterans Integrated Service Networks (VISNs) (V. H. Administration, n.d.). VISN 10 includes the lower peninsula of Michigan, Ohio, Northern Kentucky, and Indiana (Michigan, n.d.). VAAAHS is one healthcare system within VISN 10 and includes the Lieutenant Colonel Charles S. Kettles VA Medical Center (VAMC) in Ann Arbor, Michigan, and seven other community-based outpatient clinics serving a diverse patient population throughout southeast Michigan and northern Ohio (‘VA Ann Arbor Health Care,’ n.d.).

MOVE! is an evidenced-based, comprehensive lifestyle intervention supported by VA’s National Center for Health Promotion and Disease Prevention that aims to help veterans make dietary, physical activity, and behavioural modifications to support weight loss. MOVE! is delivered at all VAs in VISN 10 and consists of individual and/or group-based sessions, with in-person or virtual care options. All eligible veterans within VISN 10 may be prescribed AOMs and/or referred to bariatric surgery by healthcare professionals, including primary care clinicians. WMMHP was implemented at VAAAHS in January 2022; it is currently only available to veterans who receive care within this healthcare system. All WMMHP visits are completed virtually and individually. Bariatric surgery is available at VAAAHS and accepts referrals from all sites within VISN 10; VAAAHS’ bariatric surgery programme is one of the largest surgical weight loss programmes in the VHA with a team of dedicated surgeons and staff.

### Patients and eligibility

All veterans with a body mass index (BMI) ≥25 kg/m^2^ are eligible for MOVE! participation. WMMHP eligibility criteria align with VA’s criteria for use (CFU) for AOMs and include (1) BMI ≥ 30 kg/m^2^ or a BMI ≥ 27 kg/m^2^ combined with ≥1 weight-related medical comorbidity (e.g., hypertension, type 2 diabetes (T2D), hyperlipidaemia, obstructive sleep apnoea, osteoarthritis) and (2) participation in a comprehensive lifestyle intervention that targets three aspects of weight management, including diet, physical activity, and behaviour change (e.g., MOVE!) (‘CRE_Weight_Management_Medications_Guidance_Rev_Jan_2024.Pdf,’ n.d.).

For this study, participants will be VAAAHS patients with ≥ 1 WMMHP encounter with a team physician or CPP between January 1, 2022, and June 30, 2023, allowing at least 18 months of follow-up within the programme evaluation period. Controls will be a matched cohort of non-VAAAHS patients within VISN 10 who participated in MOVE! and meet WMMHP eligibility criteria.

### Team members

WMMHP programme is a multidisciplinary, team-based care model that largely utilizes existing team members from MOVE! Weight Management Program and primary care’s Patient Aligned Care Team (PACT), VHA’s patient-centred medical home (Ann-Marie Rosland *et al*., 2013). PACT includes primary care clinicians, CPPs, registered nurse care managers (RNCMs), licensed practical nurses (LPNs), and medical support assistants (MSAs).

WMMHP is co-directed by two PACT primary care physicians who are trained and certified in obesity medicine through the American Board of Obesity Medicine (ABOM) (DG, DM); the co-directors each devote two half days per week to providing WMMHP’s weight-focused visits. Additionally, they each devote one-half day per week to overseeing programme operations, including training new team members, developing Standardized Operating Procedures, conducting weekly team meetings, and developing new programme tools and resources.

At present, the WMMHP team also includes (1) four primary care providers (PCP) who are board certified in obesity medicine through ABOM with a combined total of 1.125 full-time equivalents (FTE); (2) three PACT CPPs with a combined total of 2.0 FTE; (3) two PACT RNCMs with a combined total of 1.5 FTE; (4) two LPNs with a combined total of 1.0 FTE; and (5) two MSAs with a combined total of 1.0 FTE. Based on current WMMHP staffing, our programme can accommodate approximately 1,600 new patient visits per year and approximately 4,000 follow-up visits.

### Training in obesity medicine

All WMMHP physicians are required to complete training and certification in obesity medicine through ABOM (American Board of Obesity Medicine, n.d.; Kushner 2018). ABOM offers all physicians the opportunity to become trained and certified in obesity medicine through a continuing medical education pathway consisting of 60 hours of online or in-person education and completion of a certifying examination (American Board of Obesity Medicine, n.d.; ‘CME Pathway Details – American Board of Obesity Medicine’ 2020). CPPs are required to complete VA-specific AOM training modules. Additionally, they are encouraged to complete obesity medicine training through the American Society of Health-System Pharmacists continuing education pathway (‘Weight Management Certificate’ 2023). Team physicians and CPPs participate initially in a 12-week training experience, consisting of once-weekly meetings with WMMHP’s medical directors to discuss new patient encounters. Meetings are approximately 45 minutes in duration; the aim is to accelerate clinicians’ obesity medicine knowledge acquisition and medical decision-making through case-based learning.

### Intervention

#### A. WMMHP intake appointment with LPN

Veterans meeting WMMHP eligibility criteria may be referred by healthcare providers through an EHR-based referral. The RNCMs review all referrals to confirm eligibility and then notify MSAs to schedule patients for initial intake appointments.

The initial WMMHP encounter consists of a 45-minute intake visit with an LPN using a structured note template (**Appendix 1**) to collect data on key topics, including weight history, prior WMT(s), and barriers to weight management (e.g., psychosocial factors, hunger and food cravings). The template includes questions adapted from our team’s prior work (Griauzde *et al*., 2022) and validated survey measures, including the modified Yale Food Addiction Scale (Schulte and Gearhardt, 2017).

The LPN places orders by protocol for fasting baseline labs, including a comprehensive metabolic panel, lipids, platelet count (to calculate Fibrosis-4 Index for Liver Fibrosis) (Anstee *et al*., 2024; Lee *et al*., 2021), hemoglobin A1c (HbA1c), serum insulin (to calculate Homeostatic Model Assessment for Insulin Resistance) (Matthews *et al*., 1985), vitamin D, and thyroid stimulating hormone. The LPN instructs the Veteran to have labs drawn prior to their initial WMMHP physician or CPP appointment (see Section B) and confirms veterans’ access to a home scale. If veterans do not have access to a home scale, the LPN places an EHR-based order to VAAAHS’s Prosthetics Department requesting that one be sent to the veterans’ home address. LPNs assess patients’ access to physical activity resources and may offer referrals to VAAAHS’ Whole Health exercise programmes, including Yoga and Tai Chi classes.

#### B. Initial WMMHP appointment with physician or CPPs

Following the LPN intake visit, patients are scheduled for a 60-minute weight-focused visit with a WMMHP clinician (i.e., physician or CPP). During this visit, clinicians use the LPN intake note to guide tailored discussion of individual patients’ weight histories, goals, challenges, and lifestyle habits (e.g., diet, physical activity, sleep). Clinicians review with patients the results of baseline laboratory tests, conduct a medication review to evaluate for use of medications that may contribute to weight gain or difficulty with weight loss, discuss the potential for weight loss to improve measures of cardiometabolic health, and inform patients of the medical and surgical options to support weight management; patients are encouraged to ask questions, express preferences, and develop personal goals. Patients interested the in use of an AOM are informed of treatment options, including FDA-approved AOMs for long-term use (i.e., liraglutide, semaglutide, tirzepatide, fixed-combination naltrexone/bupropion, fixed-combination phentermine/topiramate, and orlistat) and short-term use (i.e., phentermine). Consideration may also be given to off-label use of metformin, topiramate, bupropion, and naltrexone for weight management. Initial AOM treatment selection is informed by multiple factors, including patients’ preferences and comorbidities, VA CFU for AOMs, and AOM availability, which may vary due to national shortages (Whitley, Trujillo, and Neumiller 2023). Additionally, prior work suggests that AOMs may be more effective if selected to address the factors that drive individual patients’ eating behaviours (Acosta et al. 2021). Accordingly, initial intake measures of hunger, food cravings, and emotional eating (**Appendix 1**) may also be used to guide AOM selection. Patients eligible for and interested in the option of bariatric surgery may be referred to VAAAHS’s bariatric surgery programme for additional information and evaluation.

Patients prescribed AOMs are counselled regarding potential risks, side effects, and benefits and treatment plan expectations, including ongoing participation in lifestyle change.

#### C. Follow-up visits

We use a team-based, collaborative approach with EHR-based communication to minimize the frequency of WMMHP clinician visits while optimizing patient outcomes. Patients prescribed incretin mimetic for weight management (i.e., semaglutide or tirzepatide) are scheduled for a group-based appointment with an RNCM who provides instruction on medication administration and reviews potential side effects and strategies to mitigate them (Gorgojo-Martínez *et al*., 2022). Patients prescribed incretin mimetics for weight management are contacted monthly via phone or patient portal messaging by an RNCM until they reach a tolerated and effective dose. Patients prescribed non-incretin mimetic AOMs are contacted within 6 weeks of AOM initiation. RNCMs use a structured note template to collect the following information: (1) current weight; (2) side effects, if any; (3) desire to continue the AOM; and (4) desire to increase the AOM dose, if clinically indicated.

Follow-up visits with WMMHP clinicians are 30 minutes in duration and generally occur every 4–6 months until patients achieve weight and/or health goals. Patients are then discharged back to primary care for ongoing prescription of the AOM.

#### D. Clinical documentation

We utilize a note-writing tool created at VAAAHS through a collaboration between VAAAHS’s Software Innovation Hub and a private company (Avicenna Medical Systems, Inc.). The tool is VA-protected intellectual property that is licensed to Avicenna Medical Systems by the National VA Technology Transfer Office (TTO) (‘VA Technology Transfer Program’ 2024). The tool is commercially available within the VA health system as well as health systems outside the VA. The note-writing tool enhances clinical productivity and efficiency through several key features. First, it identifies and displays real-time patient data (e.g., weight, BMI, percent weight change, laboratory test results, prescribed medications) from VA healthcare systems. Second, the tool enables development of note templates that can be shared among team members to ensure consistent data collection, documentation, and programme fidelity. The use of templates optimizes patient safety by ensuring all patients are screened for specific AOM contraindications and precautions and counselled regarding AOM side effects. Third, within the tool, components of prior team notes (e.g., LPN new patient intake note) can be integrated and edited in subsequent notes by other providers; this minimizes duplicative patient interview questions and documentation and allows time to explore patients’ unique weight management needs, preferences, and histories. Fourth, data entered is used to generate clinical notes in a standardized format for EHR-based documentation.

## Outcome measures

### Clinical outcomes

#### Primary outcome

The primary outcome will be the mean change in weight in kilograms at 18 months compared to baseline weight. For WMMHP participants, baseline weight will be defined as the weight recorded closest to the first WMMHP encounter, that is, baseline date, among weights within −90 to +7 days. For matched controls, the baseline date will be the day of the first recorded weight in the calendar quarter in which they were matched. Weight at 18 months will be operationalized as the weight recorded closest to 18 months after baseline, limited to weights within +/−90 days.

#### Secondary outcomes

Secondary weight change outcomes will include (1) mean weight loss at 6, 12, and 24 months; (2) percent weight loss at 6, 12, 18, and 24 months; and (3) achievement of ≥5%, ≥10%, and ≥15% weight loss at 6, 12, 18, and 24 months. Outcome weights will be the weight measured closest to each of the 6-, 12-, 18-, and 24-month timepoints; timepoints without a weight within +/−90 days will be excluded. If the proportion of veterans with missing weights significantly differs between WMMHP and matched controls for any timepoint, based on a chi-square test at 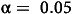 , we will impute missing weights using multiple imputation via chained equations (Van Buuren et al. 2006).

Additional secondary clinical outcomes will include (1) initial prescriptions for AOMs, (2) number of AOM prescription refills as a measure of adherence, (3) referrals to bariatric surgery, (4) engagement with bariatric surgery, defined as completing ≥1 bariatric surgery visit during the study period, and (5) completed bariatric surgeries. Initial prescriptions for AOMs will be defined as at least one order for an AOM with FDA approval during the programme evaluation period. To account for off-label prescribing, we will also include exposure to metformin, topiramate, bupropion, and naltrexone. We will also include exposure to sodium-glucose co-transporter 2 inhibitors and stimulants (e.g., methylphenidate) in our analyses, as these therapies can support weight loss.

We will examine between-group differences in measures of cardiometabolic health (e.g., lipids) using laboratory results obtained during the study period as part of routine clinical care. We will also examine health system resource utilization, including primary care visits, emergency department visits, and inpatient hospitalizations.

In sensitivity analyses, we will evaluate outcomes based on treatment type (e.g., specific AOM, bariatric surgery).

### Implementation outcomes

Implementation outcomes will include measures of programme feasibility: (1) rate of referral to WMMHP (i.e., the number of WMMHP-referred patients divided by the number of WMMHP-eligible patients), (2) uptake (i.e., the number of patients who completed a WMMHP visit with a team physician or CPP divided by the number referred), and (3) engagement (i.e., mean number of visits with WMMHP over 24 months). Implementation outcomes will be examined longitudinally during the study period.

#### Matched cohort analysis

##### Baseline characteristics

Using VA EHR data, we will identify all patients with MOVE! encounters within the VISN and subsequently eligible for WMMHP. We will extract patients’ height, weight, BMI, diagnoses for weight-related conditions, and demographic variables, including age, sex, zip code, and self-reported race and ethnicity, from the VA EHR and databases. We will identify area-level social determinants of health based on zip code.

##### Propensity matching

Among patients eligible for WMMHP with prior MOVE! participation at VAAAHS, we will model the propensity for WMMHP participation. The propensity model will be a longitudinal, discrete survival analysis implemented using logistic regression, with time discretized based on calendar year quarters. Specifically, using patient characteristics as of the date of first recorded weight in each quarter (ending March 30, June 30, September 30, and December 31), we will model the probability of WMMHP participation during that quarter among patients eligible for WMMHP. Eligible patients enter the risk pool for the propensity model in the first quarter after their initial MOVE! encounter. Independent variables for the propensity model will include baseline characteristics, as described above, percent weight change between initial MOVE! encounter and baseline, and changes in weight-related conditions during the study period. The propensity model will also allow for time-varying intercepts and coefficients to reflect both growth in WMMHP capacity and the potential for changes in referral patterns to WMMHP during the study period. The final specification of the propensity model will be determined using the Akaike Information Criterion (Akaike 1974).

We will propensity match WMMHP patients to eligible controls from non-VAAAHS sites within VISN 10 in a 1:5 match using a calliper of 0.1. Matching will be stratified by sex, quarter, and T2D status. We will assess the balance of the matched cohort by calculating standardized mean differences for baseline variables included in the propensity model (Stuart 2010).

##### Statistical analysis

We will compute descriptive statistics for baseline characteristics, including means and the interquartile range for continuous variables and counts and proportions for categorical variables. We will calculate *p*-values for baseline characteristics using ANOVA for continuous variables and chi-square tests for categorical variables.

Among the matched cohort, we will model longitudinal weight change using a linear mixed model with patient-level Gaussian random effects. The model will include binary indicators for each timepoint (6, 12, 18, and 24 months), a binary intervention effect, interactions between each timepoint and the intervention effect, and the stratification variables sex, quarter, and baseline T2D status. The primary outcome of mean change in kilograms at 18 months will be assessed by examining the coefficient and associated Wald test for the intervention x 18-month interaction, which estimates the between-group difference of the average within-person change in weight from baseline to 18 months. Secondary outcomes for mean weight change will be assessed using the other interactions. We will estimate percent weight change using the above model with log weights.

To assess achievement of ≥5%, ≥10%, and ≥15% weight loss at each timepoint, we will construct indicators for these targets by comparing weights at these targets to baseline weight. We will then use a series of univariate log-binomial regressions to estimate the relative risk at each timepoint for each weight-loss threshold. For outcomes with <5 occurrences for either WMMHP or matched controls, we will use Firth’s bias-reduced logistic regression, a method for analysing binary outcomes with a small number of observations (Firth, 1993).

We will calculate rates of referral to bariatric surgery and prescription of AOMs occurring between baseline and the 12-, 18-, and 24-month timepoints using logistic regression as previously described. We will summarize differences using odds ratio and average marginal effects (Onukwugha, Bergtold, and Jain 2015).

We will compare health system utilization outcomes, for example, primary care visits, emergency department visits, and inpatient hospitalizations, during the first 18 months after baseline using negative binomial regression with an indicator for WMMHP versus control. We will account for patients with less than 18 months of follow-up using an offset for the length of follow-up. We will summarize differences using rate ratios and average marginal effects.

## Discussion

We describe the design, rationale, and planned evaluation for a quality improvement initiative to advance obesity care delivery to veterans. Our proposed WMMHP leverages existing MOVE! and PACT infrastructure and consists of three key components: (1) weight-focused visits with physicians or CPPs trained in obesity medicine; (2) patient-centred use of AOMs and/or referral to bariatric surgery in conjunction with lifestyle change; and (3) coordinated, team-based care.

WMMHP aligns with several VA clinical and research missions. First, consistent with 2016 VHA SOTA Conference recommendations for weight management (Semla *et al*., 2017), we developed a system-level intervention to help patients successfully navigate the full range of WMT options. Second, our aim to tailor WMT plans based on veterans’ preferences supports VHA’s Whole Health model and commitment to ensuring veterans’ care is congruent with their individual values, needs, and goals (‘Whole Health Home,’ n.d.). Third, our planned WMMHP evaluation will advance VA Health Services Research and Development priorities for improving primary care and complex chronic conditions through ‘interdisciplinary collaborations…that provide patient-driven, proactive, personalized, team-based care to improve Veteran satisfaction and health care outcomes while improving costs’ (‘PriorityDomains.Pdf,’ n.d.). The ability to advance VA research missions within the context of a carefully designed clinical programme is resource-efficient, highly pragmatic, and particularly valuable given the rapidly changing landscape of obesity medicine.

To our knowledge, this is the first VA medical weight management programme to provide an in-depth description of its clinical operations and planned programme evaluation. While several prior studies have described favourable clinical outcomes from site-specific medical weight management programmes (Haverkamp, Newberry, and Baker 2022; Ni et al. 2024; Pendse et al. 2021), little is known about the staffing requirements, training procedures, visit cadence, and clinical capacities of these programmes. In contrast, we detail our programme’s infrastructure and operations, which we anticipate will support approximately 1,600 new patient visits and 4,000 return patient visits in fiscal year 2025. At present, VA weight management guidelines (VA/DoD 2020) and Pharmacy Benefits Management Services’ guidance on use of AOMs (‘CRE_Weight_Management_Medications_Guidance_Rev_Aug_2024.Pdf,’ n.d.) are nationally issued but interpreted and implemented by individual VAs. Thus, some sites may have weight management policies and procedures that are more restrictive than others (e.g., limits on prescribing of AOMs by PCPs). If our programme is effective, the details of its infrastructure and operations may guide national and local efforts to standardize obesity care delivery across VAMCs.

A recent retrospective cohort study demonstrated that only 1.6% of 534,581 eligible veterans nationwide initiated an AOM between fiscal years 2008 and 2017, though all FDA-approved AOMs were offered as a VA pharmacy benefit during the study period (Hung et al. 2024). These findings predate the availability of semaglutide and tirzepatide for weight management and may underestimate current AOM prescribing patterns within VA medical centres. Nevertheless, historically low AOM prescribing rates reveal a missed opportunity to improve individual and population health through the use of effective WMTs. For example, half of patients treated with phentermine/topiramate can achieve and sustain ≥10% weight loss (Gadde et al. 2011), a threshold associated with significant health benefits (Ryan and Yockey 2017) and reduced health care costs (Cawley et al. 2015). Yet, these benefits have not been realized due to the lack of strategies to systematically support veterans’ engagement in and successful weight loss with WMT(s). Moreover, the underuse of older, less expensive AOMs has permitted skyrocketing prescribing of new, expensive incretin mimetics for weight management (‘Obesity and GLP-1 Drugs’ 2025), which may not be necessary for or preferred by individual patients (Do et al. 2024). Accordingly, our WMMHP model aims to support a weight-centric (root cause) approach to chronic disease management while also guiding judicious use of incretin mimetics. We will evaluate healthcare utilization in our matched cohort analyses to understand potential cost implications of this approach.

WMMHP offers a promising and highly scalable approach to enhance patient-centred use of medical and surgical weight-loss treatment options. Additionally, we aim to further refine WMMHP’s efficiency and weight-loss effectiveness using two complementary strategies. First, we plan to use software to support population health weight management, including an interactive dashboard that displays real-time weight change data for all WMMHP patients and enables team-based communication. Dashboard data will guide tailored, proactive outreach by RNCMs or other team members to patients with loss-to-follow-up or non-achievement of weight change goals (e.g., <5% weight loss). In this way, limited staffing resources can be directed to patients who may need additional support, with the frequency of WMMHP return visits systematically tailored to patients’ needs. Second, diverse eating patterns can support patients’ weight loss. Yet, the MOVE! Curriculum primarily teaches veterans to follow a calorie-restricted diet (Rogers, n.d.) and there are few alternative dietary interventions offered at scale within VHA. Evidence-based alternatives such as low-carbohydrate diets, which allow for consumption of meat, cheese, nuts, and other higher-fat foods without explicit calorie restriction, may be a particularly appealing option for many veterans (Yancy et al. 2015). We are thus developing interventions to support use of carbohydrate-restricted eating patterns to enhance our menu of preference-sensitive WMT options.

## Limitations

This programme has several potential limitations. First, it will be implemented at a single healthcare system and thus may not be readily generalizable to all practice settings. However, its key components, including weight-focused visits with primary care team members trained in obesity medicine, have been successfully implemented in non-VA settings (Griauzde et al. 2024). Second, although we aim to deliver preference-sensitive WMT, factors such as national drug shortages may lead to the selection of non-preferred treatment options. Third, due to the non-randomized nature of the intervention, there may be between-group differences in patients’ desire or willingness to engage in WMT(s); these constructs are not captured in the EHR and cannot be accounted for through propensity matching.

## Conclusion

Obesity is a leading threat to veterans’ health. VHA offers robust WMT options, including a lifestyle change programme (MOVE!), AOMs, and bariatric surgery. Yet, all treatments remain underused. Despite calls to enhance delivery of effective WMT to veterans with obesity, little is known about how to do so. WMMHP offers a resource-efficient approach that may inform treatment and outcomes for patients with obesity in VA medical centres nationwide, thus filling a widespread gap in care for veterans with the potential to inform improved obesity care delivery for the greater US population.

## Supporting information

Malhotra et al. supplementary material Malhotra et al. supplementary material

## Data Availability

No new data were generated or analysed in support of this research.

## Supplementary material

The supplementary material for this article can be found at https://doi.org/10.1017/S1463423625100650.
