# Supplementary material for: Developing a Weight Management and Metabolic Health Program to support patient-centred, effective, and efficient treatment for veterans with overweight or obesity: protocol for a quality improvement programme
Source: Prim Health Care Res Dev. 2026 Jan 12;27:e9. doi: 10.1017/S1463423625100650 (PMC12817221; doi:10.1017/S1463423625100650)
Supplement: Malhotra et al. supplementary material [file S1463423625100650sup001.docx]

**Appendix 1** Structured note template for LPN Intake visit

_____________________________________________________________________________________

**PAST MEDICAL HISTORY**

_____________________________________________________________________________________

**WEIGHT HISTORY**

The patient provides the following weight history:

Childhood weight was above average/below average/average ___

Weight when patient entered the military: ___ pounds; year ___

Weight when patient left the military: ___ pounds; year ___

Current weight: ___ pounds

Peak weight: ___ pounds & year ___

Goal weight: ___ pounds

Prior weight loss medication trial

- Medication name ___

- Effectiveness ___

- Duration ___

- Adverse reaction ___

Prior weight loss diet trials

- Name ___

- Experience with the diet (free text) ___

____________________________________________________________________________

**LIFESTYLE (DIET AND PHYSICAL ACTIVITY)**

- Estimated calorie consumption: ___

- Estimated percentage of diet that is processed food (food items that is not fresh vegetables, fruits and meats/fish): ___

- Late night eating (after 7 PM): ___ # days/ week

- Water intake: ___ ounces

- Sugary beverages (type and frequency): ___

- Fast food per week: ___

- Eating out per week other than fast food:

- Food insecurity and family budget for food is ___ a concern.

24 Hour diet recall

- Breakfast: ___

- Lunch: ___

- Dinner: ___

- Snacks: ___

- Beverages: [ask about sweetened beverages, soda, juice, milk] ___

Current exercise habits: ___

- Daily steps: ___

- Strength training weekly:___

-Other

_____________________________________________________________________________________

**MOTIVATION**

Patient's motivation for weight loss is ___

- Improve health: ___

- Prevent disease: ___

- Body image: ___

- Become eligible for a surgery (like hip replacement): ___

- Self-rated motivation for weight loss (Scale of 1-10): ___

- Self-rated assessment of success with the weight loss program (Scale of 1-10): ___

- Self-rated willingness to make dietary changes (Scale of 1-10): ___

- Self-related willingness to make exercise changes (Scale of 1-10): ___

_____________________________________________________________________________________

**BARRIERS**

- To what extent do food cravings (i.e., intense desire for specific food(s)) drive your eating patterns (please answer on a scale of 1-10 where 1= “not at all” and 10= “extremely”) ___

- To what extent do high hunger levels drive your eating patterns? (please answer on a scale of 1-10 where 1= “not at all” and 10= “extremely”) ___

- Emotional eating: ___

- Eating due to being bored: ___

- Financial insecurity: Y/N, if Y specify ___

- Limited social support: Y/N, if Y specify ___

- Other: ___

_____________________________________________________________________________________

**MODIEFIED YALE FOOD ADDICTION SURVERY (mYFAS)**

*Schulte EM, Gearhardt AN. Development of the Modified Yale Food Addiction Scale Version 2.0. Eur Eat Disord Rev J Eat Disord Assoc. 2017;25(4):302-308. doi:10.1002/erv.2515*

Please respond to the statements below with ONE of the following response options:

0= Never

1= Less than monthly

2= Once a month

3= 2-3 times a month

4= Once a week

5= 2-3 times a week

6= 4-6 times a week

7= Every day

IN THE PAST 12 MONTHS:

1. I ate to the point where I felt physically ill.

2. I spent a lot of time feeling sluggish or tired from overeating.

3. I avoided work, school or social activities because I was afraid I would overeat there.

4. If I had emotional problems because I hadn’t eaten certain foods, I would

eat those foods to feel better.

5. My eating behavior caused me a lot of distress.

6. I had significant problems in my life because of food and eating. These may have been problems with my daily routine, work, school, friends, family, or health.

7. My overeating got in the way of me taking care of my family or doing household chores.

8. I kept eating in the same way even though my eating caused emotional problems.

9. Eating the same amount of food did not give me as much enjoyment as it used to.

10. I had such strong urges to eat certain foods that I couldn’t think of anything else.

11. I tried and failed to cut down on or stop eating certain foods.

12. I was so distracted by eating that I could have been hurt (e.g., when driving a car, crossing the street, operating machinery).

13. My friends or family were worried about how much I overate.

_____________________________________________________________________________________

**RELEVANT MEDICAL HISTORY**

# Hyperthyroidism [ ] Yes [ ] No

# Recent cardiac event or stroke including unstable angina (within past 6 months) [ ] Yes [ ] No

# History of gallstones within past 6 months [ ] Yes [ ] No

# History of pancreatitis [ ] Yes [ ] No

# History of severe gastrointestinal motility issues including gastroparesis [ ] Yes [ ] No

# History of chronic malabsorption syndrome or chronic diarrhea [ ] Yes [ ] No

# History of cholestasis, hyperoxaluria [ ] Yes [ ] No

# History of uncontrolled HTN [ ] Yes [ ] No

# History of nephrolithiasis (kidney stones) [ ] Yes [ ] No

# History of hypertriglyceridemia [ ] Yes [ ] No

# History of glaucoma [ ] Yes [ ] No

# History of seizures [ ] Yes [ ] No

# History of bulimia or anorexia nervosa [ ] Yes [ ] No

# History of suicide attempts or active suicidal ideation [ ] Yes [ ] No

# ESRD on dialysis, [ ] Yes [ ] No

# Severe liver impairment [ ] Yes [ ] No

# History of Type 1 diabetes [ ] Yes [ ] No

# Personal or family Hx of medullary thyroid cancer [ ] Yes [ ] No

# Personal or family history of Multiple Endocrine Neoplasia Syndrome type 2 (MENS2) [ ] Yes [ ] No

# History of alcohol abuse [ ] Yes [ ] No

# History of drug (amphetamine) abuse [ ] Yes [ ] No

# Current opioid use (prescription or nonprescription) [ ] Yes [ ] No

# Recreational drug use (if yes, list specific types) [ ] Yes [ ] No

# Current use of weight loss medications/supplements [ ] Yes [ ] No

_____________________________________________________________________________________

**SOCIAL HISTORY AND SOCIAL SUPPORT**

Patient lives with ___ and has a ___ support system at home.

His/her mood is ___ and S/he is motivated about losing weight.

S/He drinks ___ drinks per week. S/He does ___ smoke. S/He does ___ do any other drugs.

S/He currently works as a ___
